# pH Homeodynamics and Male Fertility: A Coordinated Regulation of Acid-Based Balance during Sperm Journey to Fertilization

**DOI:** 10.3390/biom14060685

**Published:** 2024-06-12

**Authors:** Pengyuan Dai, Meng Zou, Ziyi Cai, Xuhui Zeng, Xiaoning Zhang, Min Liang

**Affiliations:** Institute of Reproductive Medicine, Medical School, Nantong University, Nantong 226019, China; pengyuandai@ntu.edu.cn (P.D.); 2331310068@stmail.ntu.edu.cn (M.Z.); 2331310059@stmail.ntu.edu.cn (Z.C.); zengxuhui@ntu.edu.cn (X.Z.)

**Keywords:** pH regulation, HCO_3_^−^, H^+^, spermatogenesis, sperm maturation, regulation of sperm function

## Abstract

pH homeostasis is crucial for spermatogenesis, sperm maturation, sperm physiological function, and fertilization in mammals. HCO_3_^−^ and H^+^ are the most significant factors involved in regulating pH homeostasis in the male reproductive system. Multiple pH-regulating transporters and ion channels localize in the testis, epididymis, and spermatozoa, such as HCO_3_^−^ transporters (solute carrier family 4 and solute carrier family 26 transporters), carbonic anhydrases, and H^+^-transport channels and enzymes (e.g., Na^+^-H^+^ exchangers, monocarboxylate transporters, H^+^-ATPases, and voltage-gated proton channels). Hormone-mediated signals impose an influence on the production of some HCO_3_^−^ or H^+^ transporters, such as NBCe1, SLC4A2, MCT4, etc. Additionally, ion channels including sperm-specific cationic channels for Ca^2+^ (CatSper) and K^+^ (SLO3) are directly or indirectly regulated by pH, exerting specific actions on spermatozoa. The slightly alkaline testicular pH is conducive to spermatogenesis, whereas the epididymis’s low HCO_3_^−^ concentration and acidic lumen are favorable for sperm maturation and storage. Spermatozoa pH increases substantially after being fused with seminal fluid to enhance motility. In the female reproductive tract, sperm are subjected to increasing concentrations of HCO_3_^−^ in the uterine and fallopian tube, causing a rise in the intracellular pH (pH_i_) of spermatozoa, leading to hyperpolarization of sperm plasma membranes, capacitation, hyperactivation, acrosome reaction, and ultimately fertilization. The physiological regulation initiated by SLC26A3, SLC26A8, NHA1, sNHE, and CFTR localized in sperm is proven for certain to be involved in male fertility. This review intends to present the key factors and characteristics of pH_i_ regulation in the testes, efferent duct, epididymis, seminal fluid, and female reproductive tract, as well as the associated mechanisms during the sperm journey to fertilization, proposing insights into outstanding subjects and future research trends.

## 1. Introduction

**Figure 1:**
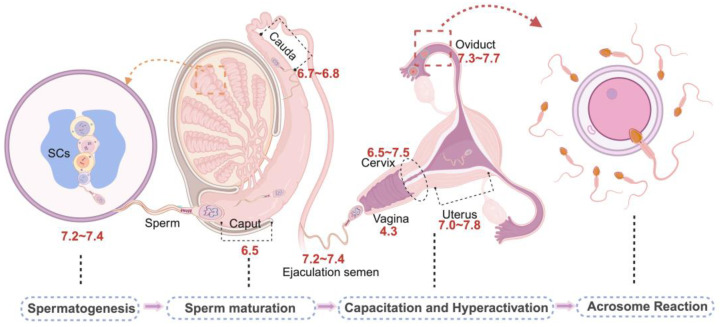
The graphical representation of pH variation in male and female reproductive systems. pH in the testis is determined at 7.2~7.4, which is conducive to maintaining spermatogenesis. From the initial segment to the cauda epididymis, the pH is approximately 6.5~6.8, where the sperm gradually matures and finally enters the vas deferens with a pH of around 7.2~7.4. After ejaculation, from the vagina to the cervix, the pH gradually alkalinizes, increasing from 4.3 to 6.5~7.5; pH 7.0~7.8 is found in the uterus, and in such an alkaline environment, sperm is capacitated and hyperactivated. pH 7.3~7.7 is maintained in the fallopian tube and contributes to AR in sperm to further fertilization.

Infertility affects approximately 15% of couples worldwide [1], with male infertility accounting for approximately 50% of these cases [2,3]. Several factors contribute to male infertility, including idiopathic abnormalities of sperm quality [4], infection and inflammation of the reproductive tract [5], genetic factors [6], and sperm ion channel dysfunction [7]. Spermatogenesis, sperm maturation, storage, and fertilization require stringent regulation of the internal microenvironment [8,9]. First, spermatogenesis is a complex process of cellular transformation in which spermatogonia undergo two meiotic divisions to form spermatozoa. In the epididymis, spermatozoa become motile and gain the ability to fertilize. Mature sperm then migrate to the vas deferens and its ampulla for storage until ejaculation [10,11]. Throughout these processes, the components of the luminal fluid, e.g., H^+^, Cl^−^, Na^+^, and HCO_3_^−^, keep changing [12], and dynamic adjustments of these ions and pH maintenance are critical for male fertility. An acidic pH (6.6–6.8) in the male reproductive tract reduces sperm motility and facilitates their maturation and storage [13]; after being deposited in the vagina, they come in contact with cervical mucus before proceeding to enter the uterus. The contraction of the uterine muscles facilitates the movement of spermatozoa to the fallopian tubes. Spermatozoa move toward the periphery of the oocyte via thermotropism and chemotropism, before sperm-egg fusion [14]. The ions present notable alterations throughout this process, leading to significant variations in the extracellular pH encountered by spermatozoa. The pH in the female reproductive tract forms a gradient, gradually increasing from the vagina (approximately pH 4.3) to the cervix (pH 6.5–7.5) and further increasing in the fallopian tubes [15]. In addition, the alkaline seminal fluid can neutralize the acidic pH of the vagina, increasing the vaginal pH from 4.3 to 7.2 [16] (Figure 1). Notably, alkalinization of the intracellular pH (pH_i_) of spermatozoa is required for sperm capacitation in mammals as well as for hyperactivation of sperm motility, chemotaxis, the acrosome reaction (AR), and fertilization [17,18]. HCO_3_^−^ and H^+^ transport are critical processes in pH_i_ regulation [19]. pH homeostasis in the male reproductive tract is managed primarily by the transporters solute carrier 4 (SLC4) and solute carrier 26 (SLC26), Na^+^-H^+^ exchangers (NHEs), carbonic anhydrases (CAs), monocarboxylate transporters (MCTs), and hydrogen voltage-gated channels (HVCN1), as well as by other channels and enzymes associated with pH regulation, including Ca^2+^ channels (CatSper) [20], K^+^ channels (SLO3) [21], and soluble adenylate cyclase (sAC) [22]. A few pH regulators in the testis and epididymis are under the control of the endocrine system [23,24,25]. Moreover, Sertoli cells (SCs) are the primary targets of hormonal transduction signals, contributing to the well-established testicular luminal microenvironment, and lactate transportation to germ cells that is metabolized from glucose in SCs [26]. Evidence suggests NBCe1, SLC4A2 [25], MCT4 [27], and MCT2 [24] levels in SCs are correlated strongly with estrogen, testosterone, follicle-stimulating hormone (FSH), and insulin. Importantly, the actions of pH regulation in male fertility are supported by multiple clinical infertility symptoms, such as asthenozoospermia, teratozoospermia, and by the aberrant sperm physiological functions of rodent sperm. A few pH regulators that are localized in the male reproductive system with determined phenotype or clinical symptoms are summarized in Table 1. This review provides a comprehensive overview of critical molecules that regulate intracellular and extracellular pH before fertilization and investigates the mechanisms responsible for pH homeostasis within microenvironments.

## 2. pH Homeostasis on Spermatogenesis

Spermatogenesis initiates with the differentiation of spermatogonia into spermatocytes, which undergo meiosis to form round spermatids, ultimately developing into spermatozoa by spermiogenesis [57]. Spermatogenesis is an orchestrating process that requires a highly specialized testicular microenvironment. In particular, HCO_3_^−^ and H^+^ play a vital role in testicular pH_i_ homeostasis [58]. SLC4 and MCTs are primarily covered in the regulation of pH_i_ in the testis.

The SLC4 family participates in HCO_3_^−^ transport and consists of 10 members, namely the Cl^−^/HCO_3_^−^ transporters (SLC4A1-AE1, SLC4A2-AE2, SLC4A3-AE3), Na^+^/HCO_3_^−^ transporters (SLC4A4-NBCe1, SLC4A5-NBCe2, SLC4A7-NBCn1, SLC4A10-NBCn2, SLC4A8-NDCBE), SLC4A9 (AE4), and SLC4A11 (BTR1) [59]. SCs play a vital role in pH_i_ regulation in the testes [60]. Multiple HCO_3_^−^ transporters are expressed in SCs, including NDCBE, NBCe1, NBCn1, and SLC4A2, which regulate pH_i_ in the testis [25,61]. NDCBE is localized in the apical plasma membrane of SCs, whereas NBCe1 and NBCn1 are found in the basal plasma membrane. NDCBE transports Na^+^ and HCO_3_^−^ from the apical lumen into the cell, exchanges them with intracellular Cl^−^, and promotes cellular alkalinization. Simultaneously, NBCe1 and NBCn1 transport Na^+^ and HCO_3_^−^ from the basal region into the cell and induce cellular alkalinization [62].

Elevated estrogen has been shown to disturb the content of NBCe1, NBCn1, and NDCBE in human SCs, impairing spermatogenesis and male fertility [62]. Estrogen receptor α (ERα) deficiency leads to decreased levels of NBCe1, CA XIV, and NHE3 (a kind of Na^+^-H^+^ exchanger) in the epididymal ducts, and increased luminal pH [23]. SLC4A2 is localized in SCs [25], spermatogonia, and mature spermatozoa [28], and its mutation blocks HCO_3_^−^-controlled cAMP signaling, disrupting spermatogenesis and causing male sterility [29]. Estrogen increases SLC4A2 levels in SCs and affects HCO_3_^−^ transport in rats [25]. In contrast, AE1 and AE3 are not expressed in most male reproductive tract tissues. AE1 is expressed only in the Leydig cells of the testis, whereas AE3 is expressed in spermatogenic cells and seminal vesicle cells [63]. The physiological functions of AE1 and AE3 in spermatogenesis remain to be elucidated.

The SLC16 family consists of 14 members, commonly termed the MCT family, which play indispensable roles in nutrient transport, cellular metabolism, and pH regulation [64]. MCT1 is expressed in spermatogonia, spermatocytes, vas deferens, epididymis, and the tails of spermatozoa [30,31,65], whereas MCT2 is expressed in elongated spermatocytes and the tails of mature spermatozoa [31]. MCT1 deficiency results in altered morphology of the seminiferous tubules and SCs, decreased serum 17β-estradiol levels, and the failure of spermatogenesis [32]. In the testes, lactate and pyruvate produced by SCs are the principal energy sources for spermatogenic cells. MCT1-MCT4 transports lactate from SCs to spermatocytes and spermatozoa [66,67]. High-intensity exercise leads to impaired spermatogenesis, decreased viability of SCs and reduced MCT-4, inhibiting lactate export from SCs [68]. In mouse spermatozoa, MCT1 and MCT2 interact with CD147 to mediate the L-lactate transport to spermatogenic cells while decreasing pH_i_ [65]. In addition, MCT1 and MCT4 interact specifically with CD147, and may contribute to MCT expression on the cell surface [67]. MCT5 and MCT8 are also expressed in the mouse testis, epididymis, and epididymal spermatozoa, but the mechanism of action remains to be investigated [65]. Hormones can influence lactate transport from SCs to spermatogenic cells by modulating MCT levels, affecting energy metabolism in the testes. The exposure of SCs to high levels of testosterone and its metabolite 5α-dihydrotestosterone leads to a considerable reduction in the production of MCT4 and lactate [69]. High levels of testosterone and follicle-stimulating hormone (FSH) have been found to decrease MCT2 content [24]. In addition, MCT4 and lactate production are notably reduced in the SCs of patients with diabetes under insulin-deprivation conditions [27].

**Figure 2:**
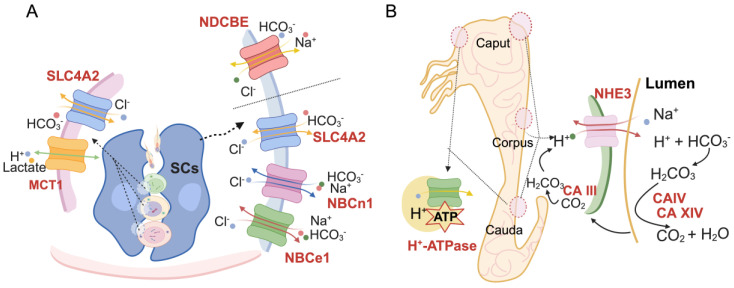
The schematic diagram presents the transport of H^+^ or HCO_3_^−^ in the testis and epididymis. (**A**) In the testis, SLC4A2 primarily transports Cl^−^ and HCO_3_^−^ in germ and SCs, and MCT1 transports H^+^. NDCBE redistributes Na^+^, Cl^−^ and HCO_3_^−^. NBCn1 and NBCe1 regulate Na^+^ and HCO_3_^−^ transportation. (**B**) In the epididymis, HCO_3_^−^ reabsorption plays a vital role in pH determination. NHE3, CA III, CA IV, and CA XIV collaborate to manage HCO_3_^−^ redistribution. In addition, H-ATPase in the caput and cauda epididymis regulates pH by participating in H^+^ secretion in the epididymal lumen.

In the testes, SLC4 and MCT transport HCO_3_^−^, H^+^, Na^+^, and lactate, stabilizing intracellular and extracellular pH within a narrow range, which is essential for maintaining spermatogenesis (Figure 2). In addition to their direct involvement in pH_i_ regulation, SCs supply lactate to germ cells [70]. Therefore, further research on lactate production and ion transport in SCs is essential to elucidate the mechanisms underlying spermatogenesis.

## 3. pH Involvement in Sperm Maturation

Sperm matures in the epididymis and acquires motility and the ability to fertilize. The epididymis is divided into four parts based on histological, structural, and functional characteristics: the initial segment, caput, corpus, and cauda [57]. As the fluid passes through the rete testis to the epididymal lumen, dramatic changes in composition, including Na^+^, Cl^−^, and HCO_3_^−^ reabsorption, H^+^ secretion, and luminal acidification are observed [12]. The acidic microenvironment of the epididymal luminal fluid and the low concentration of HCO_3_^−^ are foundational for sperm maturation [71].

The acidic lumen of the epididymis is achieved primarily through HCO_3_^−^ reabsorption and H^+^ efflux [13,72]. In the epididymis, NHE3 [41], SLC4A2 [73], NBCe1 [74], NBCn1 [75], CAs [76], and H^+^-ATPases [77] act synergistically to maintain an acidic microenvironment. NHE3 is expressed throughout the epididymis of rats and is essential for epididymal fluid reabsorption and pH regulation [38]. NHE3 knockout mice exhibited an accumulation of epididymal fluid and abnormal morphological differentiation of epithelial cells, ultimately leading to male sterility [40]. CAs catalyze the reversible hydration of CO_2_ to H_2_CO_3_, thus contributing to the maintenance of intracellular HCO_3_^−^ concentrations, which in turn influence the viability of spermatozoa. They are vital enzymes that maintain the acid-base balance of fluids [78]. The CA inhibitor acetazolamide significantly inhibits the acidification of epididymal luminal fluid [79]. The significant processes of HCO_3_^−^ reabsorption in the epididymis include the secretion of H^+^ into the lumen by the NHE3 transporter and the simultaneous reabsorption of Na^+^ from the lumen into the principal cells. H^+^ ions in the lumen react with HCO_3_^−^ to form H_2_CO_3_, while CA IV and CA XIV catalyze to form CO_2_ and water. CO_2_ enters the principal cells and is hydrated by CA III in the cytoplasm to form H_2_CO_3_, rapidly breaking down into H^+^ and HCO_3_^−^. H^+^ is then recycled into the lumen via NHE3 (Figure 2). HCO_3_^−^ diffuses passively across the basolateral side of the basement membrane via NBCe1 or SLC4A2 [40,80,81]. ERα regulates NHE3 and CAs in the epididymis. ERα knockout mice have reduced or absent NHE3 and CAs, resulting in increased epididymis luminal pH, decreased sperm viability, and male sterility [23,40]. In addition to CA III, CA IV and CA XIV, CA II, CA IX, and CA XII are also expressed in the epididymis, regulating the pH of the epididymal luminal fluid synergetically [80,81,82]. However, the underlying mechanisms are waiting to be revealed. Moreover, CA IX and CA XII are expressed on the basolateral plasma membrane of efferent duct epithelial cells and mediate transmembrane transport of Cl^−^, Na^+^ and water reabsorption in the lumen [82]. CA-mediated transmembrane ion transport is coupled to SLC-regulated HCO_3_^−^ transport, implying that CAs and SLC cooperatively regulate pH homeostasis in the luminal fluid of the efferent ducts. In conclusion, CAs are potential male reproductive pH homeostasis regulators, which further promote sperm maturation in conjunction with the HCO_3_^−^ transporter.

H^+^-ATPase is expressed in the initial segment of the epididymis, in the narrow cells in the caput, and the clear cells in the cauda [51,52,53]; it is involved dominantly in the secretion of H^+^ into the lumen of the epididymis and maintenance of the acidic pH of the epididymal luminal fluid (Figure 2) [52,83]. H^+^-ATPase exists as two subunits, B1 and B2. Mice with a knockout of the B1 subunit (*Atp6v1b1*) produce significantly more alkaline urine and are less capable of acid metabolism [84]. The FOXI1 mediates the synthesis of ATP6V1B1 in the mouse epididymis. Knockout of *Foxi1* blocks epididymal sperm maturation, disturbing fertilization due to insufficient sperm in the female reproductive tract [56]. Cadmium, a heavy metal environmental pollutant, has been shown to accumulate in the epididymis and kidneys [85], affecting fertility by inhibiting H^+^-ATPase, leading to alkalinization of the epididymal tubular lumen [86,87]. In addition, functional coordination between principal cells and clear cells in H^+^ secretion has been determined. HCO_3_^−^ is secreted by the principal cells into the lumen of the epididymis, enters the clear cells under the regulation of NBCn1, which in turn triggers sAC activation and increased cAMP levels, and finally accelerates the rate of clear cell H^+^-ATPase-mediated H^+^ secretion [88]. Angiotensin II induces the production of nitric oxide (NO), which activates soluble guanylate cyclase (sGC) in the clear cells, elevating cGMP, which in turn leads to the aggregation of H^+^-ATPase in the microvilli of the clear cells. These results indicate that epididymal H^+^-ATPase is essential to the maintenance of the acidity of the epididymal lumen and plays a crucial role in sperm maturation.

H^+^ channels and HCO_3_^−^ transporters located in epithelial cells of the epididymis, and the collaborative actions among various epithelial cell types, are essential for luminal pH regulation. Therefore, investigation of new transporters and H^+^ secretion channels and elucidation of their synergistic roles are indispensable for understanding the physiological mechanisms of sperm maturation.

## 4. pH and Regulation of Sperm Function

### 4.1. pH-Regulated Ion Channels in Spermatozoa

**Figure 3:**
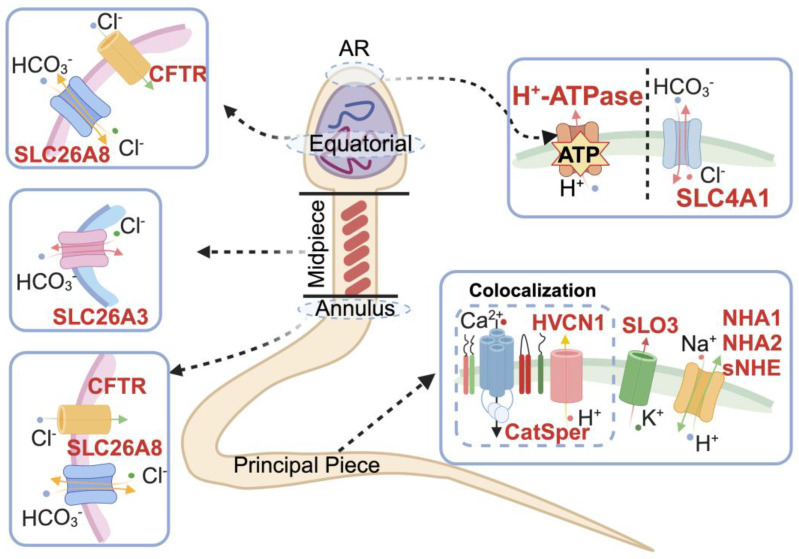
The various transporters and ion channels cooperatively regulate the pH in the sperm plasma membrane. In the acrosome of the sperm head, H^+^-ATPase is present to regulate H^+^ secretion, and SLC4A1 is involved in HCO_3_^−^ transport. In equatorial and midpieces, SLC26A3, SLC26A8 and cystic fibrosis transmembrane conductance regulator (CFTR) are found to participate in Cl^−^ and HCO_3_^−^ transport, in which SLC26A8 and CFTR are co-localized. In the principal piece of the flagellum, NHA1, NHA2, sNHE, and HVCN1 are involved in the regulation of H^+^ expulsion, among which HVCN1 is only present in human, bovine and pig spermatozoa. In addition, two sperm-specific cation channels, CatSper and SLO3, are located in the principal piece and activated by pH alkalization, mediating external calcium inflow and K^+^ expulsion, respectively. The co-localization of CatSper and HVCN1 is revealed.

As mature spermatozoa are specialized cells that are transcriptional and translational silencing, the regulation of sperm function is dependent to a large extent on ion channels, second messengers, and protein modifications [89]. Among these, the regulation of intracellular and extracellular ion concentrations by ion channels is crucial for the maintenance of sperm motility and the regulation of sperm function. pH is an indispensable regulator in the activation of ion channels. A few ion channels are present in the sperm plasma membrane, including CatSper, SLO3, voltage-gated Ca^2+^ channels, and transient receptor potential cation channel subfamily V member 4, which respond to the surroundings in the female reproductive tract [90]. Alkalinization of sperm pH_i_ activates the CatSper and SLO3 channels substantially [91,92]. CatSper is a tetrameric channel composed of four independent pore-forming α-subunits (CatSper1–4) and at least nine auxiliary subunits; it is activated by membrane depolarization and alkalinization of pH_i_, and mediates Ca^2+^ influx [93,94]. A comparative genomic assay found that nearly all animals possessing at least four CatSper pore-forming subunit genes also possessed both sAC and at least one sperm-specific Na^+^/H^+^ exchanger (*SLC9C*) gene [95], showing that CatSper activation is possibly associated with NHE activity. The external conditions appear markedly changed once spermatozoa enter the vagina. In particular, the increased pH in the fallopian tube fluid activates the CatSper channel, inducing hyperactivated motility. Male mice with knockout of any CatSper channel subunits are sterile [96,97,98]. The pH sensitivity of CatSper was initially attributed to a conserved histidine-rich region at the N-terminus of CatSper1 in mammals [91,99]. Further studies have revealed that the mechanism by which alkalinization activates CatSper is dependent on the testis-specific protein EF-hand calcium-binding domain protein 9 (EFCAB9), which directly interacts with CATSPERζ in a Ca^2+^-dependent manner and dissociates at elevated pH. EFCAB9 is significant for pH-dependent and Ca^2+^-sensitive CatSper channel activation, as CatSper is less sensitive to intracellular Ca^2+^ changes and pH_i_ alkalinization without EFCAB9 [100]. In human spermatozoa, the H^+^ channel HVCN1 co-localizes with CatSper in the principal piece of the flagellum. H^+^ efflux occurs through the HVCN1 channel, which induces pH alkalinization in the flagellum and activates the CatSper ion channel (Figure 3) [101].

SLO3 channels are pH_i_- and voltage-sensitive K^+^, which primarily mediate outward K^+^ currents (I_KSper_) (Figure 3). I_KSper_ is the only detectable hyperpolarizing current in spermatozoa and significantly regulates their resting membrane potential. *Slo3* mutant male mice are sterile, and their sperm have a meager fertilization rate in in vitro fertilization [21]. The voltage sensitivity of heterologously expressed SLO3 channels differed from that of I_KSper_, implying additional regulatory elements in the spermatozoa [92,102]. The biophysical properties of SLO3 in mice were only similar to those of natural mouse I_KSper_ when coexpressed with the auxiliary subunit LRRC52. Subsequently, LRRC52 was demonstrated to be the auxiliary subunit required to activate the SLO3 channel at physiological voltages and pH values [103,104]. Mice with *Lrrc52* gene deficiency produce spermatozoa with severely reduced I_KSper_ voltage sensitivity and have low male fertility. The standard fertilization potential of mouse spermatozoa depends on the co-expression of SLO3/LRRC52 [105]. Intracellular alkalinization significantly enhances I_KSper_, but this effect can be inhibited by the NHE inhibitor EIPA [92], indicating that I_KSper_ is regulated by H^+^. In addition, when extracellular pH was at a level of 7.4 and there was no pH buffer in the pipette solution, dimethyl amiloride (DMA), a broad-spectrum NHE inhibitor, inhibited KSper current and membrane depolarization, and subsequently led to a decrease in CatSper current and Ca^2+^ influx [106], further demonstrating the pH-sensitive regulation of SLO3.

Possible links in functional coupling between pH-sensitive CatSper and SLO3 have been found; however, species differences have led to contradictory results among studies. Evidence from mouse spermatozoa suggests that SLO3 controls Ca^2+^ influx through CatSper [107]. High HCO_3_^−^ concentration causes changes in pH_i_ that activate SLO3 channels [108]. The membrane hyperpolarization further raises pH_i_ via NHE or HCO_3_^−^ transporters, and this pH_i_ alkalinization activates CatSper channels, leading to a rapid increase in [Ca^2+^]_i_ [107]. In human spermatozoa, KSper is regulated by Ca^2+^ but is not sensitive to pH [109]. SLO3 channels can inhibit Ca^2+^ influx via CatSper, proving it may be localized downstream of CatSper [110]; this suggestion is enhanced by the investigation in which some ICSI patients exhibited impaired K^+^ conductance and abnormal resting membrane potentials but normal resting [Ca^2+^]_i_ and P4-induced [Ca^2+^]_i_ responses that were similar to those of normal spermatozoa, suggesting that CatSper function was not altered [111]. Additionally, inhibitor cross-sensitization between CatSper and SLO3 has been revealed. Therefore, further studies are required to investigate the potential interactions between these two channels, with a particular emphasis on elucidating the role of pH in the functional coupling between them, and identifying the similarities and differences between mouse and human spermatozoa. In addition, the emphasis should be on whether other ion channels present in spermatozoa, such as Cl^−^, Na^+^, and other Ca^2+^ channels, are modulated by pH and affect sperm function.

### 4.2. pH and Sperm Motility

The initiation of sperm motility in fish, birds, and mammals has shown that although pH affects sperm motility to slightly differing extents or in subtly different ways, it plays a crucial regulatory role in initiating or maintaining sperm motility, and alkalinization is generally considered to facilitate these processes [112]. After maturation, spermatozoa are stored in the epididymis in an immotile form. In addition to being constrained by high osmotic pressure and the need for various proteins in the spermatozoa to be functionally integrated and separated, the lower pH may block their motility. For example, the motor proteins that drive the movement of the sperm flagellum are pH-dependent, and a lower pH inhibits dynein ATPase activity [113]. This was also observed in an assay concentrating on sea urchin spermatozoa [20]. After ejaculation, sperm motility is initiated by combining factors such as hypotonicity, extracellular Na^+^, K^+^ levels, and pH [114,115].

The extracellular pH of the spermatozoa increases instantaneously to approximately 7.5 in seminal fluid. pH value increase contributes to sperm motility, and activation of sperm motility by alkalinization, and was even once believed to be conserved from corals to humans [116]. Alkalinization stimulates cAMP production by sAC, which leads to protein kinase A (PKA)-dependent phosphorylation of flagellin and opening of CatSper channels, promoting sperm motility [117,118]. The elevated pH is critical for activating sperm motility, and this process depends on extracellular Na^+^ mediated by the activation of the Na^+^/H^+^ exchanger in Pacific oysters [119]. However, intracellular alkalinization does not participate in sperm motility in marine fishes such as the European eel [120], and pH_i_ remains unchanged in the sperm motility of rainbow trout [121], implying that species should be taken into consideration in uncovering the pH-mediated process.

In addition to direct regulation of sperm pH_i_ by intracellular factors, the external pH can regulate sperm motility by affecting pH_i_. The motility was significantly higher in alkaline culture conditions (pH 7.2 and 8.2) compared to that in acidic conditions (pH 5.2 and 6.2) in human spermatozoa, which may be related to the reduced concentration of intracellular Ca^2+^ in the spermatozoa and the inhibition of Na^+^/K^+^-ATPase activity under acidic conditions [122]. Clinical investigations have also found that the pH of seminal plasma in some cases with oligozoospermia and asthenozoospermia is significantly lower than that of the general population (pH 7.2) and that pH is positively correlated with sperm motility [123]. Therefore, alkalinity is essential for sperm motility in vitro. Sperm motility in vivo is significantly activated when they arrive at the glucose-rich uterus from the lactate-rich vagina, demonstrating that glucose is a pH-dependent regulator of sperm motility and that the production of HCO_3_^−^ from glycolysis is responsible for elevated pH_i_ [124]. Sperm viability decreases during fluid preservation of livestock spermatozoa, accompanied by reduced pH. Artificial pH stabilization is essential for maintaining sperm viability during fluid conservation [125]. Cryopreserved spermatozoa are also associated with decreased pH after resuscitation, which is detrimental to their survival. Therefore, the development of non-toxic pH buffers or diluents that contribute to the recovery of spermatozoa after resuscitation is necessary. In addition, sperm frozen at a higher pH (8.0) have significantly enhanced viability after resuscitation [126], which can be applied to improve the assisted reproduction rate. These data suggest that acidification of the culture medium or seminal fluid after activation of sperm motility is detrimental to normal sperm motility and survival.

Taking advantage of the role of pH in regulating sperm motility, researchers have attempted to selectively screen or enrich sperm with different sex chromosomes (X or Y) through pH manipulation to generate sex-specific offspring in animal husbandry and veterinary medicine [127]. Studies employing human and bovine spermatozoa showed no difference in the response of X- and Y-chromosome spermatozoa to changes in pH. However, spermatozoa from dairy goats in diluents with different pH levels had different proportions of upper-layer X/Y-chromosome spermatozoa using a swim-up technique. At a sperm diluent pH of 6.2, the proportion of upper layer X-chromosome spermatozoa was 67.24% ± 2.61. At a sperm diluent pH of 7.4, the proportion of upper layer X-chromosome spermatozoa was 30.45% ± 1.03, which differed significantly from a proportion of 52.35% ± 1.72 in the control group (pH 6.8) [127]. A higher proportion of X-chromosome spermatozoa (85.57% ± 3.27) was observed in the alkaline diluent (pH 7.4) upon the co-administration of resiquimod [128]. pH affects mitochondrial activity and glucose uptake capacity primarily through phosphorylation of NFκB and GSK3α/β. The viability of X-chromosome spermatozoa is inhibited under acidic conditions and significantly enhanced under alkaline conditions [129]. The sex ratio of offspring can be influenced by adjusting the pH of the semen diluent in pigs, with female offspring more likely to be obtained from semen stored under acidic conditions for short periods (<1 d) [130,131].

### 4.3. pH Promotion in Sperm Capacitation and Hyperactivation

After entering the female reproductive tract, the progressively more alkaline pH and high HCO_3_^−^ concentration induce capacitation of the sperm cells. During capacitation, physiological-biochemical variation is observed in spermatozoa, including pH_i_ alkalinization, increased intracellular Ca^2+^ ([Ca^2+^]_i_) levels, plasma membrane hyperpolarization, and tyrosine phosphorylation. These processes are critical for sperm capacitation and hyperactivated motility, further facilitating AR and fertilization [132]. Hyperactivated motility accompanies the onset of capacitation and manifests as high-amplitude, asymmetric oscillation of the sperm flagellum, which enables the sperm’s arrival at the fallopian tube. Alkaline pH plays a double role in hyperactivation. First, intracellular alkalinization promotes sperm hyperactivated motility by activating CatSper channels [133]. In mice, pH_i_ is elevated during sperm capacitation, which activates CatSper channels and triggers asymmetric bending of the flagellum to stimulate hyperactivated motility [134]. Treatment with the alkalizer NH_4_Cl increases pH_i_, stimulates elevated sperm [Ca^2+^]_i_, and induces hyperactivation [135]. Second, pH alkalinization directly induces hyperactivated motility. The spermatozoa without membrane could be reactivated in solutions containing high concentrations of ATP and 400–1000 nM Ca^2+^, leading to asymmetric oscillation of their flagella [136,137]. In addition, demembranated spermatozoa were activated at pH 7.0. However, hyperactivated motility occurred only in solutions at pH 7.9–8.5, suggesting that an increase in pH in the axonemal compartment of the spermatozoa is conducive to hyperactivated motility [137].

pH_i_ alkalinization is required for sperm capacitation and largely depends on HCO_3_^−^ influx or H^+^ efflux [47,138]. High HCO_3_^−^ concentration in the female reproductive tract is crucial for the cAMP-PKA signaling pathway, pH_i_ alkalinization, and further membrane hyperpolarization [22,139,140]. HCO_3_^−^ regulates adenylate cyclase (sAC or ADCY10) activity in the spermatozoa and induces increased cAMP levels and activation of PKA. Male mice with knockout of both sAC genes are sterile, with spermatozoa lacking progressive motility and exhibiting inhibited pH_i_ alkalinization, affecting both sperm capacitation and hyperactivated motility [118,141,142]. In addition, the sAC inhibitor TDI-10229 also significantly inhibits pH_i_ alkalinization, increased cAMP content, and AR induced by sperm capacitation in humans and mice [143].

#### 4.3.1. HCO_3_^−^ Transport

In spermatozoa, HCO_3_^−^ transport is mediated through the SLC26 family and CFTR (Figure 3). The central SLC26 family members localized in the male reproductive tract and spermatozoa include SLC26A3, SLC26A6, and SLC26A8 (TAT1) [36,144]. In mouse spermatozoa, SLC26A3 and SLC26A6 are expressed primarily in the midpiece. SLC26A3 is involved in increased intracellular Cl^−^ and membrane hyperpolarization induced by dibutyryl cyclic AMP (dbcAMP). Furthermore, HCO_3_^−^ is transported through SLC26A3, which plays a crucial role in capacitation and induces tyrosine phosphorylation and hyperactivated motility in spermatozoa [33,145]. Mice with *Slc26a3* knockout have altered epididymal morphology and reduced sperm counts, which resulted in male sterility [34], whereas *Slc26a6* knockout mice remained fertile [146]. A missense mutation in *Slc26a3* in human spermatozoa causes decreased male fertility [35]. SLC26A3 and SLC26A6 also interact with CFTR and function together in regulating sperm pH_i_ [33], suggesting that SLC26A3 plays a vital role in male reproduction. SLC26A8 is predominantly localized in the annulus of mature human and mouse spermatids [36,37]. In male mice, deletion of the *Slc26a8* gene results in structural anomalies such as defective flagellar differentiation, abnormal flagellar loops, and abnormal sperm capacitation, which ultimately causes male sterility [36]. Notably, SLC26A8 co-localizes with CFTR, another factor that regulates pH homeostasis by transporting HCO_3_^−^, in the equatorial segment of the sperm head (Figure 3). Together, they form a complex that participates in sperm capacitation and hyperactivated motility. SLC26A8 stimulates CFTR channel activity. Loss of *Slc26a8* in mouse spermatozoa leads to decreased intracellular cAMP, which abrogates activation of the sAC/PKA signaling pathway [147]. 

Mutations in *Cftr* cause cystic fibrosis, characterized by progressive lung disease, pancreatic insufficiency, and male infertility [49,50,148]. In mice, guinea pigs, and human spermatozoa, CFTR is localized in the equatorial region of the head and the midpiece of the sperm [47]. Homozygous *Cftr* knockout mice tend to die around puberty; therefore, heterozygous *Cftr* mutant mice (*Cftr*^+/−^) are commonly used to identify the role of CFTR in spermatozoa [48]. *Cftr*^+/−^ spermatozoa exhibited abnormal capacitation, attenuated response to HCO_3_^−^-induced membrane hyperpolarization, reduced cAMP production, and decreased motility compared to those of wild-type mice. Additionally, the CFTR inhibitor diphenylamine-2-carboxylic acid (DPC) can affect membrane hyperpolarization and inhibit Cl^−^ influx [47,149], whereas the agonist genistein induces membrane hyperpolarization and Cl^−^ influx in non-capacitated mouse spermatozoa [149]. Notably, CFTR is in the flagellar annulus of spermatozoa along with both sAC and SLC26A8, promoting local cAMP production; this may be necessary for flagellar PKA pathway activation in spermatozoa [147]. Besides, CFTR can modulate hyperpolarization associated with sperm capacitation in mice by inhibiting epithelial Na^+^ channels, which activate the cAMP/PKA signaling pathway and regulate membrane potential [150,151].

#### 4.3.2. H^+^ Transport

H^+^ efflux is mediated primarily through H^+^ channels on the sperm plasma membrane, which induces pH_i_ alkalinization and elevated cAMP levels (Figure 3), facilitating sperm capacitation. Sperm pH_i_ alkalinization was abnormal and capacitation was inhibited after H^+^ efflux blocking [106,152,153]. In rodents, H^+^ efflux during capacitation is mainly dependent on NHEs. Inhibition of NHE channels significantly reduces sperm viability and progressive motility [106]. NHEs are a class of Na^+^/H^+^ exchangers involved in the electroneutral exchange of extracellular Na^+^ with intracellular H^+^ to keep pH homeostasis. The NHE family consists of 13 members organized into three distinct subfamilies: (1) SLC9A: SLC9A1(NHE1)–SLC9A9(NHE9); (2) SLC9B: SLC9B1(NHA1) and SLC9B2(NHA2); (3) SLC9C: SLC9C1 (sperm-specific NHE exchanger, sNHE) and SLC9C2 [154]. NHEs regulate the acid-base balance of a wide range of luminal fluids. Several NHEs have been detected in the male reproductive tract or spermatozoa, including NHE1, NHE2, NHE3, NHE5, NHE8, NHA1, NHA2, and sNHE [39]. 

The localization, molecular weight, and physiological actions of NHE1 vary among species. In rats, NHE1 is localized in the midpiece of the flagellum and cooperates with the a4 isoform of Na^+^/K^+^-ATPase to regulate pH_i_ and maintain sperm motility [155]. In sheep and pig spermatozoa, NHE1 is localized in the equatorial segment of the head and flagellum and regulates sperm viability and pH [156]. The absence of NHE1 or NHE5 does not affect fertility in male mice [157,158]. 

NHE2 is localized in the caput, corpus, and cauda of the epididymis, and in the vas deferens and testes [159,160]; however, limited information is available regarding its function. NHE3 is expressed in the efferent ducts and proximal epididymis in rats and is localized to the efferent ducts in humans and mice, where it is involved in the reabsorption of Na^+^ and in regulating luminal fluid pH [38,39]. 

Knockdown of *Nhe3* results in abnormal dilation of the rete testis lumen and efferent ducts, leading to obstructive azoospermia [39]. In spermatogenic cells, NHE3 is localized explicitly to developing acrosomal granules, and its absence results in severe defects in the acrosome, indicating that NHE3 is essential for normal acrosome development [40].

*Nhe8* knockout mice exhibit impaired spermatogenesis, impaired testicular Leydig cell function, round-headed spermatozoa, lack of acrosomes, and abnormal distribution of mitochondrial sheaths [61]. Mice with SC-specific knockdown of *NHE8* produce spermatozoa with normal morphology and exhibit no adverse effects on fertility [161]. In addition, NHE8 is not expressed in Leydig cells [161]. Evidence showed that NHE8 is essential for male fertility; however, its expression and physiological functions in the SCs and Leydig cells, and its working in acrosome formation, remain to be elucidated. 

NHA1 and NHA2 are specifically localized to the principal piece of the mouse sperm flagellum (Figure 3) [42]. *Nha1* and *Nha2* cKO mice exhibit decreased sperm counts and reduced fertility. In addition, the spermatozoa of *Nha1* cKO mice have markedly reduced levels of cAMP synthesized by sAC [42]. *Nha1/2* double knockout mice showed male sterility [42]. In humans, NHA1 deficiency has been reported in men with teratozoospermia [43]. 

sNHE is localized in the principal piece of the sperm flagellum [44] and is sensitive to voltage-gating and cAMP (Figure 3) [45]. In mice, sNHE is coupled to the SLC26A3-CFTR complex, facilitating high levels of HCO_3_^−^ and Cl^−^, thus ensuring elevated cAMP and pH_i_ alkalization [33]. Deficiency or loss of function of *sNhe* leads to infertility in mice, primarily due to reduced HCO_3_^−^-sensitive sAC activity, resulting in lower cAMP content and impaired sperm hyperactivation [44,162]. Decreased sNHE was also detected in patients with asthenozoospermia, and the level of sNHE was positively correlated with sperm concentration, total number, and progressive motility [46]. sNHE regulates the pH_i_ of the sperm flagellum, and is involved in sperm motility, functionally coupling with CatSper and Ksper [153]. NHE11 is localized in the acrosomal region of the mature mouse and human sperm [163]; however, its physiological role has not been elucidated.

The HVCN1 is mainly localized in the principal piece of the flagellum as a critical player in the control of pH_i_ of spermatozoa. In human, bovine, and porcine spermatozoa, HVCN1 also mediates H^+^ efflux besides NHEs (Figure 3) [101]. The activation of HVCN1 depends on membrane potential and the difference between intracellular and extracellular pH. Additionally, fatty acids regulate the HVCN1 gating via the protein kinase C (PKC)-dependent phosphorylation pathway [164,165,166,167]. During sperm capacitation in human or bovine models, HVCN1 induces rapid alkalinization of the principal piece of the flagellum, activating CatSper channels and pH-dependent proteins that contribute to the transition of spermatozoa into motility patterns [168,169]. HVCN1 was found to regulate pH_i_ alkalinization and the hyperactivation of motility in the principal piece of the flagellum in human spermatozoa after inhibition by Cl-GBI [152]. In porcine sperm, HVCN1 is essential for the hyperactivation of motility during capacitation in vitro, but does not influence the luteinizing hormone-induced AR [138]. In addition, eliminating cholesterol and high levels of albumin in the uterine fluid can also enhance the activation of HVCN1 channels [170,171]. Notably, low levels of albumin in the semen (approximately 15 µM) cannot activate HVCN1 channels. In comparison, albumin (500 µM) in the female reproductive tract causes HVCN1 channels to open and induces sperm capacitation, enabling fertilization [170,172]. In contrast, high levels of Zn^2+^ in human seminal fluid inactivate HVCN1 and reduce sperm viability [173].

### 4.4. Acrosome Reaction

In mammals, the acrosome is a secretory vesicle located in the apical region of the sperm head, and the acrosome releases its contents by exocytosis upon physiological stimulation, a process known as the AR [174]. The acrosome contains a mixture of hydrolytic enzymes promoting passage of the sperm through the oocyte for sperm-egg fusion [175]. Mouse sperm acrosomal pH (pH_a_) is maintained at 5.3 but increases to approximately 6.2 during capacitation [176]. Alkalinization during acrosome capacitation activates various enzymes within the acrosome and stimulates spontaneous AR [175,176,177]. Inhibition of lactate metabolism results in an inability to alkalinize pH_i_, which further affects normal capacitation and the occurrence of AR [178]. In addition, elevated pH_a_ promotes the breakdown of amyloid in the acrosomal matrix amyloid and facilitates the exocytosis of the acrosomal contents [179]. The percentage of AR induction was highest when spermatozoa were at a pH of 7.4, whereas an acidic extracellular condition (pH 6.5) inhibited AR [180,181]. In human spermatozoa, pH_a_ gradually increases when incubated in capacitation fluid, depending on the presence of HCO_3_^−^ and Ca^2+^ in the medium, and alkalinization of the acrosome is mediated by a combination of H^+^-ATPase, HCO_3_^−^ transporter, and sAC (Figure 3) [182]. A weakly alkaline Ca^2+^ blocker induces acrosomal alkalinization, triggering AR in mouse and human spermatozoa, even in a calcium-free medium. NH_4_Cl, a well-known alkalizer, failed to induce AR, suggesting that Ca^2+^ release from the acrosomes is a critical process in the induction of AR. However, alkalinization alone cannot induce AR [183,184]. Inhibition of H^+^-ATPase leads to acrosome alkalinization in mice and humans, possibly by maintaining and generating an H^+^ gradient in the acrosome membrane, which regulates pH_a_ [54]. However, alkalinization caused by H^+^-ATPase inhibition cannot induce AR, indicating that acrosomal alkalinization and AR may be relatively independent processes or that alkalinization is unnecessary for AR [55]. This may differ from AR in sea urchin spermatozoa, where alkalinization is believed to be required for AR [20].

The subject of how pH regulates AR is largely unknown, and it is currently thought to involve alterations in enzyme activity and the activations of various ion channels associated with H^+^ and HCO_3_^−^ transport. SLC4A1, which is expressed in the head and flagellum of human spermatozoa, is essential for the initiation of AR and may be related to the regulation of phosphorylation of two specific protein kinases in the sperm head, namely the tyrosine kinases Syk (which phosphorylates Tyr8, Tyr21, and Tyr904) and Lyn (which phosphorylates Tyr359) [185].

SLC26A8 acts as an anion transporter and may directly regulate AR via pH-dependent activation [36] or functional coupling with CFTR [186]. Inhibition of CFTR with the channel blocker inh-172 also markedly inhibits mouse spermatozoa from AR and affects HCO_3_^−^-induced alkalinization and membrane hyperpolarization, suppressing the HCO_3_^−^-dependent increase in cAMP [47]. These results imply that CFTR promotes AR depending on pH variation. In addition, NHE1, another necessary channel regulating pH, was found to be localized in the equatorial segment of the sperm head and the flagellum in porcine spermatozoa [187]. NHE1 is essential for progesterone-induced hyperactivation and AR [187]. Interestingly, reduced sNHE was found in patients with necrospermia, and its expression was also determined to be positively correlated with sperm concentration but not with the rate of AR [46]. However, our data confirmed that sNHE affects progesterone-induced AR by modulating the pH of the sperm flagellum instead of that in the acrosome [153]. HVCN1 is also a considerable channel in AR activation by coupling with cAMP, PKC, or CatSper in bull spermatozoa [168]. Various ion channels are implemented in AR activation, while the associated mechanisms and the possibility of synergistic effects require further determination.

## 5. Conclusions and Perspectives

Various membrane proteins have been identified as working synergistically to sustain proper intracellular and extracellular pH by transporting HCO_3_^−^, H^+^, Na^+^, etc., contributing to spermatogenesis and sperm maturation. In the female reproductive tract, HCO_3_^−^ transporters and H^+^ channels alkalinize sperm pH_i_, inducing sperm capacitation, hyperactivated motility and AR. The significance of pH homeostasis has been explored in laboratories by effective methods such as the patch-clamp technique, voltage-sensitive and ion-sensitive dyes, bilayer reconstitution, recombinant DNA techniques, single-stranded probe (cRNA) expression in heterologous systems, immunocytochemistry, etc. Further investigations are warranted to uncover the mechanisms of the transporters and ion channels regulating the pH of the luminal fluid of the reproductive tract as well as the pH_i_ of spermatozoa. Our recommendations are as follows: 1. It remains unclear whether SLC4A1 and SLC4A3 (Table 2) are involved in pH regulation during spermatogenesis and sperm maturation. 2. Localized pH-regulating channels other than CatSper and SLO3 need further clarification. 3. Whether pH participates in regulating thermotropism and chemotropism before fertilization needs to be illuminated. 4. NBCe2, NHE2, NHE11, CA II, CA IX, MCT8, etc. (Table 2), are localized in the male reproductive system, and their involvement in male fertility needs to be identified. 

Several sperm pH regulators have been shown to be implicated in male fertility during clinical investigations through multiple complicated pathophysiologic processes, including SLC26A3, SLC26A8, NHA1, sNHE, and CFTR. Damaged synergistic action of SLC26A3 and CFTR in HCO_3_^−^ transport appeared in infertile men [35]. SLC26A8 is localized in the human sperm annulus, the aberrant expression of which caused structural defect of the annulus, further leading to asthenospermia [37]; however, the role of the annulus in sperm motility is far from clear, and the H^+^/HCO_3_^−^ transportation activity of SLC26A8 provided a novel clue in the functional exploration of the sperm annulus. Moreover, determining the influence of other SLC26 family members on spermatozoa and their cooperation mechanism with CFTR would contribute to revealing the pathogenesis of male infertility. The expression of NHA1 occurs in a DNA methylation-dependent and independent concerted manner mediated by diverse DNA regulatory elements, and the decrease or absence of NHA1 is relevant to teratozoospermia [43]. Additional studies should focus on the methylation and demethylation patterns in NHA1 CpG islands from teratozoospermic cases, to determine the action of DNA methylation in NHA1 transcription regulation, and its contribution to male reproduction [43]. sNHE deficiency is also responsible for the pathogenicity of asthenospermia, probably due to pH_i_ alkalization disorders [46]. Therefore, further clarifying the mechanisms of pH-regulating transporters involved in male fertility is crucial for targeted treatment in clinical practice. In addition, further screening infertile sperm samples in the clinic to focus on reproductive defects caused by disorders of acid-base balance, and identifying novel HCO_3_^−^ and H^+^ transporters as well as their specific functions, will contribute to enhancing our understanding of male infertility, and may lead in turn to the development of novel therapeutic strategies for patients with oligospermia, necrospermia, and idiopathic infertility.

## Figures and Tables

**Table 1:** The silence of pH regulators causes male reproductive deficiency.

Protein Symbol, Gene ID	Location	Functions	Phenotype (Knockout in Mouse)	Clinical Symptoms/Disease
SLC4A2 (AE2), 6522	Sertoli cells (SCs), spermatozoa [25,28]	Sodium-independent anion exchanger, transports Cl^−^/HCO_3_^−^ in sperm.	Spermatogenesis interrupted, male infertility [29]	NR
SLC16A1 (MCT1), 6566	Elongated spermatids, spermatozoa tail [30,31]	Bidirectional proton-coupled monocarboxylate transporter, transports nutrients, and regulates pH.	Seminiferous tubules and SCs morphologically changed, and spermatogenesis failed [32]	NR
SLC26A3, 1811	Midpiece [33]	The chloride/bicarbonate transporter, participates in energy acquisition, induces sperm tyrosine phosphorylation and is hyperactivated.	Epididymal morphology altered, and mature sperm decreased [34]	Male infertility [35]
SLC26A8 (TAT1), 116369	Annulus of spermatozoa [36,37]	Co-localization with CFTR in the equatorial segment of the sperm head, involves sperm capacitation and hyperactivation.	Sperm flagellar differentiation is defective, and sperm capacitation fails [36]	Asthenozoospermia [37]
SLC9A3 (NHE3), 6550	Efferent duct [38,39], developing acrosomal granule [40], epididymis [41]	Reabsorb Na^+^ in the efferent duct, and regulate lumen fluid pH.	The lumen of the rete testis and efferent duct dilated, causing obstructive azoospermia [39]	NR
SLC9B1 (NHA1), 150159 SLC9B2 (NHA2), 133308	Principal piece [42]	Na^+^/H^+^ exchanger, regulates the intracellular pH of spermatozoa, and maintains sperm motility and fertility.	Sperm count decreased, causing low fertility (NHA1 or NHA2 cKO mice) and male infertility (double KO mice) [42]	Teratospermia, caused by NHA1 deficiency [43]
SLC9C1 (NHE10/sNHE), 285335	Principal piece [44]	Sperm-specific sodium/hydrogen exchanger, regulates the intracellular pH of spermatozoa, and maintains sperm hyperactivation.	Male infertility and asthenozoospermia [45].	Asthenospermia [46]
CFTR, 1080	The equatorial region of the sperm head and middle piece [47]	The epithelial ion channel, maintains chloride and bicarbonate homeostasis during sperm epididymal maturation and capacitation.	Homozygotes for targeted null mutations cause death around puberty. *Cftr*^+/−^ male mice exhibited abnormal capacitation [48]	Cystic fibrosis: progressive lung disorder, pancreatic insufficiency, male infertility [49,50]
B1 subunit of the H^+^-ATPase (ATP6V1B1), 110935	The initial segment of the epididymis, narrow cells in the caput epididymis and bright cells in the cauda epididymis [51,52,53], acrosome [54,55]	Participate in the secretion of the H^+^ in epididymal lumen and maintain the acidic pH of epididymal lumen fluid, and acrosome alkalization.	FOXI1 regulates the synthesis of ATP6V1B1. *Foxi1*^−/−^ mice sperm maturation was blocked, and fertilization failed [56]	NR

NR: not reported.

**Table 2:** pH regulators in the male reproductive system deserve further investigation.

Protein Symbol, Gene ID	Location in Other Tissues	Functions	Location in the Male Reproductive System
SLC4A1 (AE1), 6521	Erythroid, kidney [188]	Electroneutral anion transporter and structural protein [188]	Leydig cells [63]
SLC4A3 (AE3), 6508	brain, heart, and adrenal gland [189,190]	Chloride ion exchangers, regulate intracellular pH and cardiac action potential [189,190]	Spermatogenic cells and seminal vesicle cells [63]
SLC4A5 (NBCe2), 57835	Liver, spleen, kidney, etc. [191]	Sodium-bicarbonate cotransporter [191]	Testis, epididymis, prostate, and seminal vesicles [192]
SLC9A2 (NHE2, ID:6549)	Kidney, stomach [160]	Plasma membrane Na^+^-H^+^ exchanger, maintains intracellular pH [160]	Epididymis, vas deferens, testis [159,160]
SLC9C2 (NHE11), 284525	Heart [193]	Sodium/hydrogen exchanger, correlates with human cardiac dysfunction, and regulates intracellular pH [193].	Spermatozoa head [163]
CA II, 760	Stomach, brain, duodenum, etc. [194,195,196,197]	Catalyzes reversible hydration, creates and maintains the pH differential in tumor cells, and regulates the pH of duodenal villous epithelial cells [194,195,196,197]	Epididymis (narrow cells of the initial segment and principal cells of all regions) [81]
CA IX, 768	Brain, duodenum, stomach, eye [195,198,199,200]	Catalyzes reversible hydration, participates in necrosis, calcification, acid-base balance, and the formation of aqueous humor, and gastric acid, and maintains pH homeostasis [198,200,201,202]	Epididymis (efferent duct) [82]
CA XII, 771	Expresses widely in normal tissues [203,204]	Reversible hydration of carbon dioxide [203], and maintains intracellular pH [204]	Epididymis [81,82]
SLC16A2 (MCT8), 6567	Brain, thyroid, kidney [205]	Specific thyroid hormone transmembrane transporter, mediating efflux of thyroid hormone across cell membranes [205]	Mouse testis, epididymis, epididymal spermatozoa [65]
SLC16A5 (MCT5), ID: 9121	Liver, kidney, brain, prostate, etc. [206]	Proton-linked monocarboxylate transporter [206]	Mouse testis, epididymis, epididymal spermatozoa [65]
